# The Influence of Competing Social and Symbolic Cues on Observers’ Gaze Behaviour

**DOI:** 10.3390/vision8020023

**Published:** 2024-04-16

**Authors:** Flora Ioannidou, Frouke Hermens

**Affiliations:** School of Psychology, University of Lincoln, Lincoln LN6 7TS, UK; ioannidouflora@gmail.com

**Keywords:** visual attention, eye movements, social cues, symbolic cues

## Abstract

The effects of social (eye gaze, pointing gestures) and symbolic (arrows) cues on observers’ attention are often studied by presenting such cues in isolation and at fixation. Here, we extend this work by embedding cues in natural scenes. Participants were presented with a single cue (Experiment 1) or a combination of cues (Experiment 2) embedded in natural scenes and were asked to ‘simply look at the images’ while their eye movements were recorded to assess the effects of the cues on (overt) attention. Single-gaze and pointing cues were fixated for longer than arrows but at the cost of shorter dwell times on the cued object. When presented together, gaze and pointing cues were fixated faster and for longer than simultaneously presented arrows. Attention to the cued object depended on the combination of cues and whether both cues were directed towards or away from the target object. Together, the findings confirm earlier observations that people attract attention more strongly than arrows but that arrows more strongly direct attention.

## 1. Introduction

Humans can shift their visual attention covertly (without moving their eyes) and overtly (by moving their eyes). Covert visual attention is commonly measured using the cueing paradigm [1,2], in which participants are presented with a peripherally (e.g., a dot left or right of fixation) or centrally presented cue (e.g., an arrow pointing left or right). After a delay, the cue is followed by a peripheral target (e.g., the letter ‘F’ or ‘T’), and participants are asked to respond as quickly and accurately as possible (e.g., by looking at it or by indicating the target’s identity by pressing the corresponding key on a keyboard). Covert attention to the target is assumed when faster responses are observed towards a cued target than towards a target that is not cued. By varying the time between the cue and the target, the time course of shifts of covert attention can be determined.

Experiments using the cueing paradigm have suggested that two types of attention shifts exist: exogenous and endogenous attention shifts [3,4]. Exogenous attention shifts are commonly found for peripherally presented cues [5,6], which is thought to be an automatically triggered, fast-acting (and fast-decaying) form of attention that also exhibits inhibition of return at longer intervals between the cue and target [7]. Endogenous attention shifts are typically found for centrally presented cues (e.g., arrows) and are thought to be under voluntary control, to take more time to build up, and to not show inhibition of return.

Around 1998, two studies appeared that suggested that social cues consisting of a centrally presented face stimulus with averted eye gaze elicited exogenous attention shifts [8,9]. Such averted-gaze stimuli, for example, led to faster responses to a gazed-at peripheral target, even when the direction of the gaze was counterpredictive of where the target was going to appear (i.e., the target more often appeared away from the direction of gaze; [8,9,10]). Eye gaze was also found to result in inhibition of return at longer cue–target intervals [11]. The exogenous attention shifts for gaze cues were at odds with the idea that exogenous attention can only be triggered by peripherally presented stimuli. This suggested that social attention (induced by gaze cues) may be more potent than attention shifts induced by other centrally presented cues, such as arrows. The results led to suggestions of an innate gaze detection module in the human brain [12,13].

A debate followed on the special nature of gaze cues. Some studies replicated the original paradigms in a direct comparison between gaze and arrow cues and found similar cueing from both types of cues [14,15,16,17,18,19,20,21]. Other studies suggested that arrows may show such behaviour because they are highly over-learned cues [21,22,23]. For example, after training with texture cues for three weeks, a similar cueing time course could be found for these texture cues as for arrows and gaze cues [21].

The cueing paradigm, while providing a highly controlled method to measure covert attention, also presents cues in a way that is unlike how cues are normally encountered. Faces are hardly seen in isolation and rarely on an otherwise empty background with nothing else to look at (other than the peripheral target or place-holders for this target). A possible further limitation of the cueing paradigm to study social attention was later noticed. The strong cueing effects of gaze cues were only found when the cues appeared at fixation and not randomly above or below fixation [24,25]. In contrast, rotated heads and pointing fingers, as well as arrows, were effective cues [25], suggesting a role for visual crowding that is stronger for a pair of eyes than for a rotated head, pointing fingers, or arrows. While the cues in these studies were presented on an otherwise empty screen, the results may have implications for viewing natural scenes because, typically, cues in such scenes do not appear where people are already looking.

Authors have therefore argued that it is important to determine the effects of gaze cues in more realistic settings [26,27] than the traditional cueing paradigm [1]. A first step towards such natural settings, which still allows for a relatively high level of control over stimulus conditions, is the use of photographs. With photographs, the same stimulus can be shown to each observer, while everything other than the cue can easily be varied. The presentation of photographs is often combined with ‘free viewing’, because tasks are known to affect eye movement patterns [28,29]. As a consequence, the natural-scene, free-viewing paradigm probes into overt and not covert attention because of the use of eye movements to investigate visual attention [27,30,31,32,33].

To compare visual attention to social and symbolic cues in natural scenes, the same scenes are presented to participants with just a change in the cue (from a person gazing, a person pointing, or an arrow pointing) while eye movements are recorded. A complicating factor in such a setup is that cues often differ in size. One of the studies therefore compared the effects of gazing people with the effects of a standing loudspeaker [33], but one could argue that a loudspeaker may not be a clear directional cue (not like an arrow sign). Direct comparisons of how often the cued object is fixated are similarly difficult, because the time available to look at the cued object will depend on how much time is spent looking at the cue itself (i.e., more time on the cue means less time available to look at the cued object). Hermens and Walker [31] therefore proposed to compare the rates of saccades from the cue (once the cue was fixated) to the cued object (in comparison to elsewhere in the scene). These rates suggested stronger cueing for pointing gestures and arrows than for a person gazing at the cued object.

Instead of presenting cues in isolation, it may therefore be beneficial to place cues in direct competition by studying gaze behaviour for natural scenes that contain two cues. If, for example, a photograph contains a person and an arrow, and observers spend significantly more time looking at the person, this suggests that the person attracted more attention than the arrow. If saccades from the arrow more often lead to fixations on the target than saccades from the person, this suggests stronger cue following for the arrow. This approach of presenting multiple cues within the same scene was adopted by Birmingham and colleagues [27], who presented six photographs that contained both an arrow and a person and found that observers often fixated the person, but not the arrow. The possible limitations of this study, however, were the limited number of images and the strong reliance on arrows that were embedded in signs also containing either text or arrows painted on the street. The study did not look at the saccades that followed fixations on the cues.

The present work therefore extends the work by Birmingham and colleagues [27] by comparing the gaze patterns of observers when presented with photographs containing no cues, a single cue, two cues in competition, and two cues in conjunction. Cues were provided by humans (either gazing or pointing at an object in the scene) or arrows. We present the results of two experiments. Experiment 1 used single cues, replicating the paradigm by Hermens and Walker [31] but correcting for possible issues with this previous study by using a larger number of actors providing the cues, a larger number of scenes, larger arrow signs that better matched the size of the people in the scene, and better-defined regions of interest (previously, the bounding boxes around pointing arms included a large section of the background). Experiment 2 then moved on to multiple cues by including images with two cues, either in competition or in conjunction. If there is an advantage of social over symbolic cues, the people in the scenes are expected to be fixated more often and for longer when they are presented together with arrows. Experiment 2 also aimed to show whether the rates of saccades from the cue to the target are higher for social than for symbolic cues.

## 2. Experiment 1

Experiment 1 replicated the paradigm by Hermens and Walker [31] but used more precisely defined regions of interest (ROIs), larger arrow signs that better matched the size of the people in the scene, a broader range of stimuli, and a larger number of actors.

### 2.1. Methods

#### 2.1.1. Participants

Thirty psychology students from the University of Lincoln (UK) participated in the study. The participants were aged 18 to 36 years (mean = 20.7 years, sd = 4.1 years), of whom 27 self-identified as female. All participants reported normal or corrected-to-normal vision. We asked them to wear contact lenses if they had these available instead of glasses to improve the quality of the eye-tracking data. The participants all provided written consent. The study was approved by the School of Psychology Ethics Committee (SOPREC) at the University of Lincoln (UK). The participants took part in return for course credit.

#### 2.1.2. Apparatus

A quiet lab in the Psychology Building of the School of Psychology at the University of Lincoln was used to collect the data. The lab hosts an Eyelink 1000 (SR Research, Ottawa, ON, Canada) desk-mounted eye tracker, which was used for eye tracking. This Eyelink 1000 eye tracker measures the gaze direction of the participant at a rate of 1000 Hz and has a reported average accuracy between 0.25° and 0.5° and a reported resolution (RMS) of 0.01°. Stimulus presentation was controlled with the SR Research Experiment Builder software. We always tracked each participant’s left eye (the IR illuminator of the eye tracker was on the right) using a combination of pupil position and corneal reflection. A 19-inch Dell flat screen (with a resolution of 1280 × 1024 pixels, which corresponds to approximately 26° × 20° of visual angle) was used to show the photographs to the participants. The screen was placed at around 80 cm from the chin rest that stabilised the participant’s head during data collection. The photographs shown to the participants were taken with a Samsung Galaxy A3 smartphone camera. This camera had a resolution of 1920 by 1080 pixels.

#### 2.1.3. Stimuli

The photographs that were shown to participants consisted of four times 19 indoor and outdoor scenes at the University of Lincoln (UK) campus and of residential areas of Athens, Greece. Each scene was photographed four times: (1) with an arrow pointing at the target object, (2) with an actor gazing at the target object, (3) with an actor pointing at the target object, (4) without a cue. The actors and arrow signs were always at the same position for each scene (but varied between scenes), as was the target object (which was typically already in the scene at that location). Ten different actors provided the gaze and pointing cues in this study (Hermens and Walker [31] had two actors). In 57% of the images, cues were placed on the left side of the image, whereas in the remaining 43% of the images, the cues were on the right. Each of the photographs was reduced in size to 800 by 600 pixels (approximately 16° × 12° of visual angle inside the 1024 by 768 pixel screen resolution). Images were always presented in the middle of the screen. This left some space around the images for the presentation of the fixation target shown before the image at one of four positions (left, right, top, or bottom [34]).

#### 2.1.4. Design

The participants saw all 76 images (19 scenes, each with four cue conditions) in a semi-random order. A design was used in which the images were presented in four blocks (unknown to the participants), with each scene presented once in each block and the cues systematically varied across blocks. Within each block, the order of the images was randomised for each participant. The blocks were varied across participants using a Latin square. The design was such that, within each block, there were equal numbers of each cue and that scenes were, on average, evenly distributed across the trial sequence.

#### 2.1.5. Procedure

The participants were asked to rest their heads in a chin-and-head rest to avoid influences of head movements on the estimated gaze direction. The standard nine-point calibration of the Eyelink system was used. Calibration was repeated until the system indicated that the calibration was ‘good’. The researcher also checked that the recorded gaze points were aligned with the nine calibration points before continuing with showing the stimuli and recording eye movements. Under these conditions, the reported resolution of the system is better than 0.01°, and the average accuracy is between 0.25° and 0.5°.

To avoid a central bias [35] by design, each trial was initiated by the experimenter when the participant fixated a drift correction target presented at one of four (randomly selected) positions outside the image (left, right, top, bottom). The drift correction target therefore had two purposes: to correct for drift in the data due to small head movements during and between trials and to avoid scenarios where participants always started fixating the middle of the image [34]. Each of the images was presented for 2000 ms, and a break was offered after every 38 images, in line with the earlier study [31]. The time between the trials depended on how long participants took to fixate the drift correction target and how long the experimenter took to confirm fixation, and therefore, it varied across trials (duration not recorded). The participants were asked to simply view the images without any further instruction. Typically, the participants had participated in other studies in the psychology department before and knew that they would be debriefed after the experiment about the purpose of the study.

#### 2.1.6. Data Analysis

**Figure 1:**
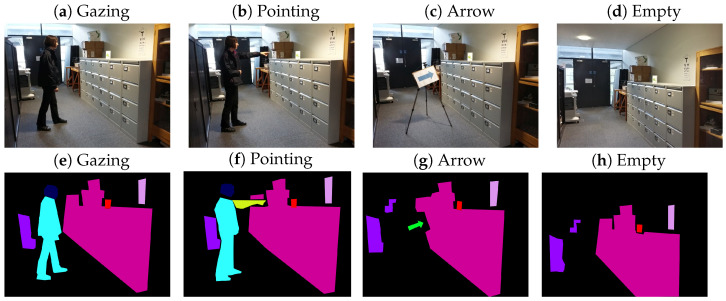
Examples of images (**a**–**d**) and regions of interest (**e**–**h**) for the four conditions for one of the scenes. For human actors, three regions of interest were used: the head, the body, and the pointing arm (only for pointing cues). In panels (**e**–**h**), cyan pixels indicate the region of the body of the person, dark-blue pixels the head of the person, yellow pixels the arm of the person, and green pixels the position of the arrow. The light-pink region indicates the target. We also coded the positions of other objects in the scene (other colours and black) but did not use these regions for the analysis (these regions were all mapped onto the ‘elsewhere’ category).

Using the default settings of Eyelink’s parser software (SR Research, Ontario, Canada), raw horizontal and vertical gaze positions on the screen were automatically parsed into fixations and saccades. Using the GIMP software packages, the images shown to the participants were converted to ROI images by adding a layer with colours for each ROI (see Figure 1). Fixations were assigned to these ROIs by superimposing them onto these images and coding the colour below each fixation. This procedure produces better-fitting ROIs compared to the bounding boxes used by Hermens and Walker [31], particularly for diagonally upward- and downward-pointing hands and arms. The ROI for an arrow was set as the region occupied by the arrow itself, but not the rest of the sign (see Figure 1).

To account for the use of multiple scenes per cue type in the statistical analysis, linear mixed-effects models were used with participants and scenes as random factors (and, for example, cue type as a fixed effect), using the *lme4* package [36] in *R*, as an alternative to ANOVAs of participant means per condition [37]. The comparison of models with and without a fixed factor of interest (or interaction) yields a chi-squared statistic that can be compared to a reference distribution.

We examined whether the results depended on the choice to use mixed-effects analyses rather than ANOVAs and *t*-tests of participant means, and we tested for differences between an analysis of the entire data set (with repeated scenes) or just the first block (with each scene presented only once—so no repetitions of scenes per participant). No obvious differences were found between the outcomes. We therefore only report mixed-effects results and analyses across all trials.

We examine the influence of the various cues on gaze behaviour by examining a combination of dwell times (on the cues and targets), the time course until fixation on the cue and the target and the distribution of directions of saccades leaving the cue. The reason for examining multiple measures is to obtain a complete picture of the effects of cues on eye movement behaviour. A possible limitation of dwell times is that when a fixed presentation duration is used (as was the case in the experiments), this means that if participants spend more time looking at the cue, they can spend less time looking at the target (their point of gaze may get stuck on the cue).

The analysis of the time course until the first fixation on the cue will inform how much time participants spend looking at other parts of scenes until they reach the cue or the target and whether they fixate the cue or target at all. Finally, the distribution of the directions of saccades leaving the cue will inform how strong the tendency is to follow the direction of the cue towards the target from the moment the cue is fixated.

### 2.2. Results

#### 2.2.1. Dwell Times

**Figure 2:**
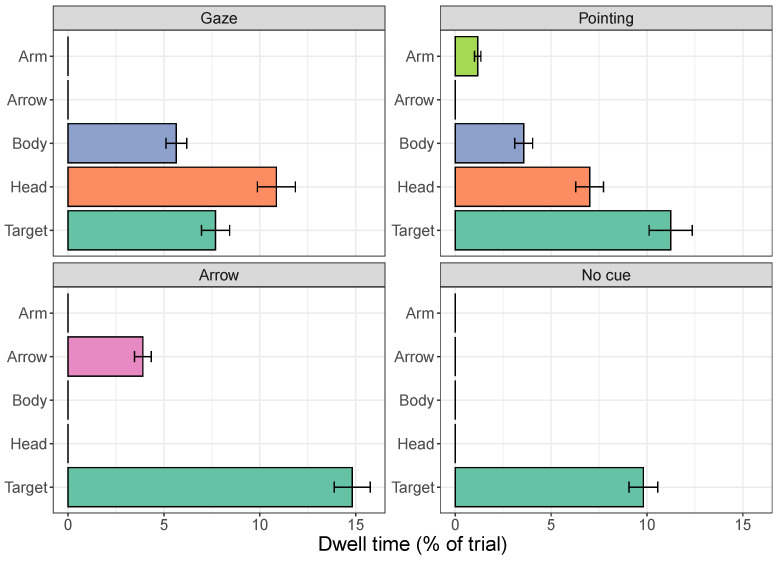
Dwell times on the different regions of the cues and the target for each of the four conditions in Experiment 1. Dwell times are shown as a percentage of the overall time spent looking at the image. Error bars show the standard error of the mean across cues.

Figure 2 plots the dwell times on each of the regions of the cues and the target as a percentage of the overall time spent looking at the image. It shows that for the gaze and pointing cues, people fixate on the head for longer than the remainder of the body. The pointing arm is fixated for relatively short amounts of time. As a whole, the gaze cue is looked at for the longest (16.5% of the trial), followed by the pointing cue (11.8% of the trial) and the arrow (3.9%). These differences in dwell times on the entire cue are statistically significant, even after adjusting the critical *p*-value for the four comparisons (all *p*-values < 0.0001).

The time spent looking at the target (we use the name ‘target’, but it was the same object in the absence of a cue) also differs across conditions. The longest dwell times on the target are found for the arrow cue (14.8%), followed by the pointing cue (11.2%), no cue (9.8%), and the gaze cue (7.7%). Statistical comparisons show significant differences in dwell times on targets when all four conditions are compared ($\chi^{2}$(1) = 73.9, p< 0.0001), when the three conditions with cues are compared ($\chi^{2}$(1) = 64.1, p< 0.0001), and when comparisons are made between arrows and gaze cues ($\chi^{2}$(1) = 66.2, p< 0.0001), between arrows and pointing cues ($\chi^{2}$(1) = 14.9, p< 0.0001), between gaze and pointing cues ($\chi^{2}$(1) = 18.4, p< 0.0001), between arrows and no cue ($\chi^{2}$(1) = 35.1, p< 0.0001), and between the gaze cue and no cue ($\chi^{2}$(1) = 8.38, *p* = 0.0038).

**Figure 3:**
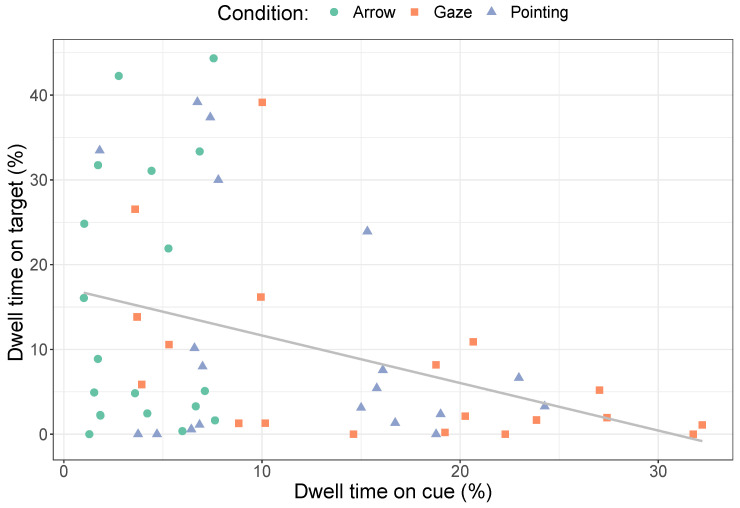
A comparison of dwell times on cues and the target. Each symbol indicates one image in the experiment. Different shapes show different conditions. The grey line shows the best-fitting regression line.

The longer dwell times on the target for the arrow and pointing cues compared to no cue suggest that the cue leads to more time spent looking at the target. At the same time, time spent on the cue may take away time that could be spent on the target. Figure 3 shows that longer dwell times on cues indeed lead to shorter dwell times on the target (*r* = −0.36, *p* = 0.0061). Note, however, that this correlation is only found when all three cues are included in the analysis. When examining cues separately, a significant correlation is only found for the gaze cues (gaze cues that are fixated for longer lead to shorter dwell times on the target, *r* = −0.51, *p* = 0.025), not for arrows (*r* = 0.096, *p* = 0.70) or pointing cues (*r* = −0.36, *p* = 0.13). The lack of significant correlations for the arrow and pointing cues may be due to a smaller range of dwell times on the cue itself and due to fewer observations overall compared to when all cues are included in the analysis.

#### 2.2.2. ROI Size and Position

**Figure 4:**
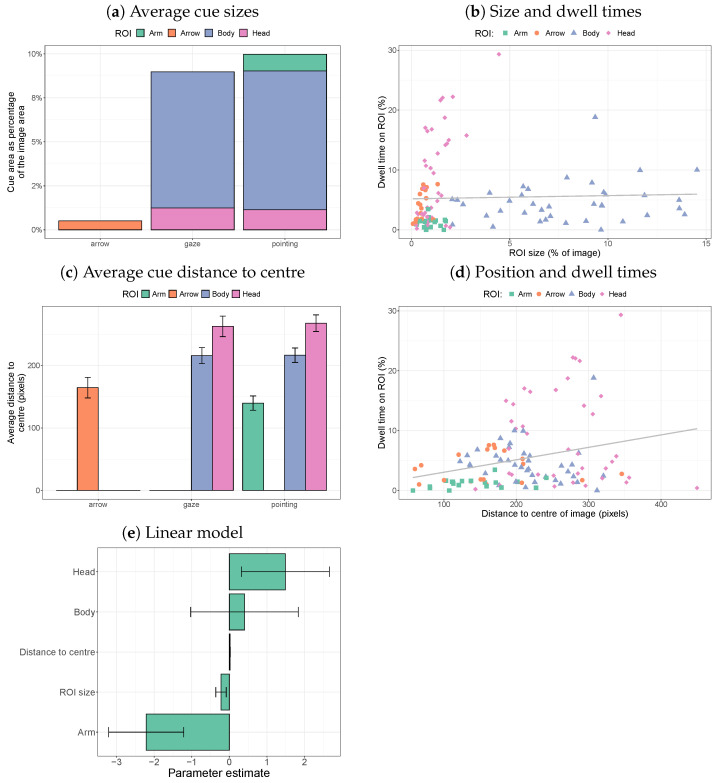
The influence of the central bias and ROI size on dwell times on ROIs. Relatively long dwell times on heads and targets are found, given their size and position in the image, whereas dwell times are lower than expected for bodies (reference category = arrow).

The different ROIs vary in size and position within the image. Larger ROIs have a higher chance of being fixated, even if participants randomly scan the image. Due to the central bias [35], ROIs closer to the centre of the image can be expected to be fixated for longer. Figure 4a shows that the social cues take up substantially larger portions of the images than the arrow cues. In particular, the bodies of the people in the scene take up space (the size difference between arrows, heads, and arms is much less).

Figure 4b does not suggest that bodies, which are substantially larger in size, have much longer dwell times. There was no significant correlation between size and dwell time across the various ROIs (*r* = 0.036, *p* = 0.70). Within cue types, there were significant correlations between size and dwell times for arrows (*r* = 0.60, *p* = 0.0068) and heads (*r* = 0.64, p< 0.001), but not for bodies (*r* = 0.20, *p* = 0.23) and arms (*r* = −0.021, *p* = 0.93).

Figure 4c plots the average distance of each of the cue pixels to the midpoint of the image, showing that arrows and arms were, on average, closer to the image centre than heads and bodies. Figure 4d shows that ROIs that were, on average, closer to the centre of the image had longer dwell times. Across ROIs, this led to a positive correlation between the distance and dwell time (*r* = 0.27, *p* = 0.0030). Within each ROI type, however, there were no significant correlations between the distance and dwell time (all *p*-values > 0.12).

The joint effects of ROI size, distance to the centre, and ROI type can be investigated by fitting a linear mixed-effects model, predicting dwell time on the basis of these three factors. The results are shown in Figure 4e. By comparing the fit of the model with one without the distance to the centre, we find that distance significantly contributes to the prediction of dwell times ($\chi^{2}$(1) = 5.37 *p* = 0.021). A similar comparison for ROI size shows that the size does not play a role in dwell times ($\chi^{2}$(1) = 1.97, *p* = 0.16). Finally, the ROI type provides a significant contribution to the prediction of dwell times ($\chi^{2}$(3) = 13.3 *p* = 0.0041). The coefficients in Figure 4e indicate that dwell times on heads are longer than expected on the basis of their size and position, and dwell times on arms are shorter than expected on the basis of their size and position.

#### 2.2.3. Time to First Fixation

Previous studies have looked at the time until the first fixation, e.g., [32]. The analysis of these times needs to take into account that participants do not always fixate on the cues or the target. We, therefore, employ a survival analysis approach, which treats trials without fixation on the cue or the target as ‘censored’ (i.e., fixation may happen after the end of the trial, but we simply do not have that information).

**Figure 5:**
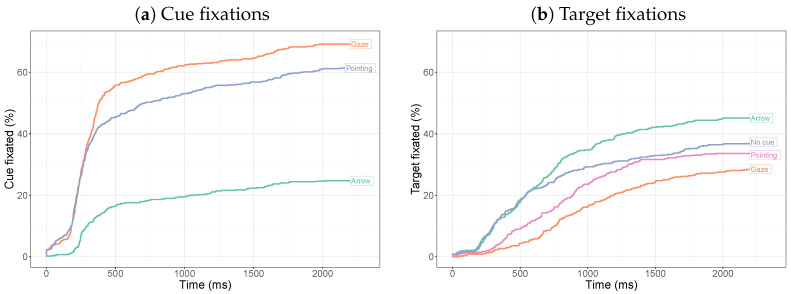
Inverse survival curves for the time until fixation on the cue or the target for the different conditions. For cue fixations, the entire cue was used (including body and arm). In constructing the curves, the individual trials were treated as independent measurements.

Figure 5 shows the time until fixation on the cue or target as a function of time. These curves are based on all trials across participants. Fixations on the social cues mostly occur early in the trial, whereas fixations on the arrow are delayed (and less frequent).

To test the difference between the three conditions, we fit mixed-effects Cox regressions and compared a model with a main effect of cue type and one without using a likelihood ratio test. There is a significant main effect of the cue ($\chi^{2}$(2) = 285.4, p< 0.001). Pairwise comparisons show significant differences between the gaze and arrow ($\chi^{2}$(1) = 249.6, p< 0.001) and between pointing and the arrow ($\chi^{2}$(1) = 174.3, p< 0.001), but not between pointing and gaze cues ($\chi^{2}$(1) = 6.54, *p* = 0.011, not significant after a Bonferroni correction).

Target fixations occur earlier for the arrow cue than for the gaze and pointing cues. The curves for no cue and the arrow cue initially follow the same trajectory and start to diverge after around 600 ms, suggesting that first fixations on the target due to the arrow cue tend to occur later. There is a significant main effect of the cue (all four cues compared: $\chi^{2}$(3) = 46.7, p< 0.001). Pairwise comparisons show a significant difference between the gaze cue and the arrow ($\chi^{2}$(1) = 44.3, p< 0.001) and between the pointing cue and the arrow ($\chi^{2}$(1) = 19.8, p< 0.001), but not between the gaze and pointing cues ($\chi^{2}$(1) = 4.97, *p* = 0.02585, not significant after a Bonferroni correction). There is a significant difference between the arrow and no cue ($\chi^{2}$(1) = 7.02, *p* = 0.0081) and between the gaze cue and no cue ($\chi^{2}$(1) = 14.0, *p* = 0.00019), but not between the pointing and no-cue conditions ($\chi^{2}$(1) = 2.70, *p* = 0.1003).

#### 2.2.4. Target-Directed Saccades

**Figure 6:**
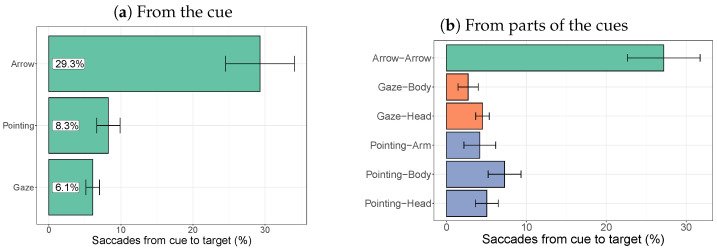
Saccades that start from the cue or parts of the cue landing on the target.

While cues differ in size, within each scene, the target is always the same. Once participants fixate the cue, the next saccade therefore provides an indication of how strongly the cue directs this saccade towards the target. Figure 6 plots the percentage of saccades that start from the cue and land on the target as a percentage of all saccades leaving the cue. It shows that arrows led to substantially more fixations on the target than the gaze and pointing cues. A comparison of the fit of mixed-effects logistic regression models showed a significant main effect of the type of cue on the chance of a subsequent fixation on the target ($\chi^{2}$(2) = 48.3, p< 0.001). Pairwise comparisons showed a significant difference between the arrow and the pointing cue ($\chi^{2}$(1) = 31.7, p< 0.001) and between the arrow and the gaze cue ($\chi^{2}$(1) = 45.9, p< 0.001), but not between the pointing and gaze cues ($\chi^{2}$(1) = 2.00, *p* = 0.16).

The same analysis can be performed for each part of the cues (arm, head, and body) by comparing the number of saccades going to the target to the number of saccades going elsewhere (another part of the cue or elsewhere in the image). This again shows the larger number of saccades leaving the arrow to the target compared to the other two cues. For the gaze cue, more saccade fixations on the head lead to more fixations on the target than fixations on the body. For the pointing cue, it is the body that leads to the most subsequent fixations on the target than the arm or head. These differences, however, are not statistically significant. If the arrow cue is removed from the analysis (i.e., when just the different regions of the bodies are compared), the other regions do not show a significant difference between them ($\chi^{2}$(4) = 7.50, *p* = 0.11).

### 2.3. Discussion

Experiment 1 used the paradigm by Hermens and Walker [31] but with a larger number of scenes, more actors, larger arrow signs, and better-defined regions of interest. It replicated some but not all of the findings from the previous study. In agreement with the previous study, arrows were looked at less than people in the scene, also in line with [27], who placed these cues in direct competition, and in line with several studies that found that people strongly attract observers’ attention [30,34,38,39,40]. In contrast to Hermens and Walker [31], who found more time on the target for pointing cues compared to arrows, Experiment 1 found more time on the target for arrows than for pointing cues or gaze cues. In fact, we found a negative correlation between the amount of time spent looking at the cue and looking at the target.

The results for the percentage of saccades leaving the cue differed from Hermens and Walker [31] as well. Substantially more target-directed saccades left the arrow cue than the pointing or gaze cues. This may be due to the larger arrow cues, which may have induced stronger cueing than the relatively small cues in Hermens and Walker [31]. The narrow ROI around the arrow may have played a role as well, as only fixations that were on the arrow itself were counted in the present analysis. Interestingly, the arm did not lead to stronger cueing than the body for the pointing cue. Outgoing saccades may provide the best evidence for attention cueing from the three measures presented. While cues differ in size and shape, the target is always the same. Once the cue is fixated, the outgoing saccade direction provides a means to determine how strongly the cue directs attention.

## 3. Experiment 2

Experiment 2 placed social and symbolic cues in direct competition (pointing at different targets) or in conjunction (pointing at the same target). It aimed to determine which type of cue is looked at more often and whether cueing effects can be combined (cues in conjunction) or weakened (cues in competition).

### 3.1. Methods

Twenty-four students (nine males, aged between 19 and 30) from the University of Lincoln (UK) participated in Experiment 2 in return for course credit. As in Experiment 1, participants all reported normal or corrected-to-normal vision. They each gave written consent for the study, which fell under the same ethics approval as Experiment 1.

**Figure 7:**
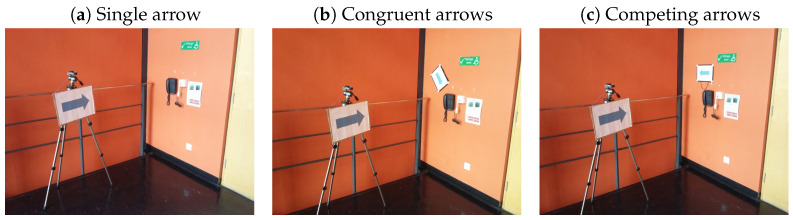
Examples of stimuli from Experiment 2. Only scenes with arrows are shown to protect the privacy of our actors. There were also scenes with congruent and competing gaze and pointing cues together with the arrow cue. The target object for this particular scene was the phone on the wall. The ‘away’ cue can be seen pointing in a different direction, but not to an object in particular. This was the case for most of the ‘away’ cues.

The same setup as for Experiment 1 was used, with the exception of the photographs shown to the participants. Participants were shown a total of 133 photographs. These consisted of seven indoor and outdoor scenes at the University of Lincoln for each of the nineteen cue combinations (see Table 1). Examples of images are shown in Figure 7). Cues towards the target always pointed to an object in the image. Cues away from the target were placed opposite to this target and often pointed towards an object outside the image. As in Experiment 1, images were scaled to a resolution of 800 by 600 pixels.

Most of the targets were presented near the vertical mid-line but at different heights in the image. Cues were often positioned to the left and right of the target, but not always, as the example in Figure 7 shows. To avoid order effects, the order of the images was pseudo-randomised so that subsequent trials did not show the same scene. As in Experiment 1, participants were asked to ‘simply view the images’, each presented for two seconds, without further instruction. Short breaks were offered after every 35 trials.

### 3.2. Results

As indicated in Table 1, there are many possible combinations of one or two cues. In the following, we will present the results grouped by the towards cue (arrow, gaze, or pointing). This means that some of the results can appear in multiple graphs, for example, when a towards cue is combined with another towards cue. By clustering the results by the towards cue, the effects of the other cue for that towards cue can be compared. As for Experiment 1, three dependent measures will be examined: dwell times (on cues and targets), saccade directions from the cue, and time until the first fixation (on the cue and the target).

#### 3.2.1. Dwell Times on Cues

**Figure 8:**
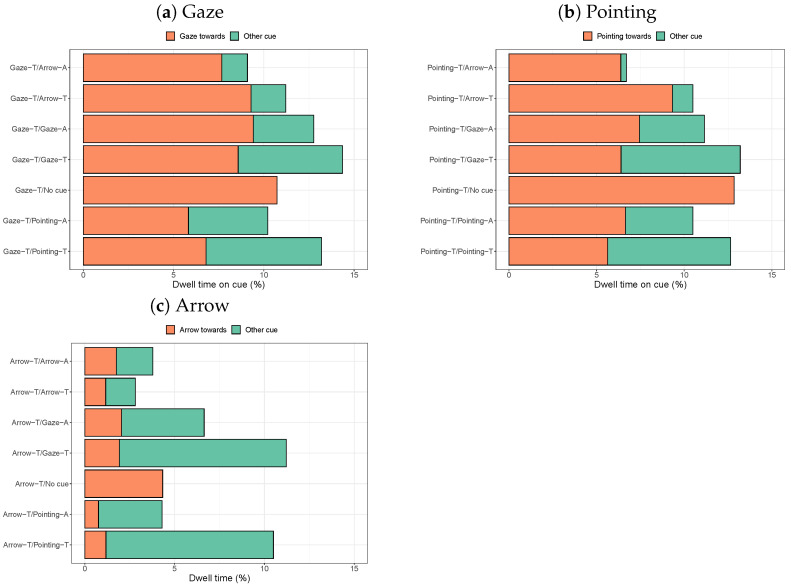
Dwell times on the cue of interest (as a percentage of the total viewing time in a trial) and on the second cue in the scene (if present).

Experiment 2 asked the question of whether gaze behaviour in the presence of one cue depends on the presence of another cue. Figure 8 compares the dwell times on the cue of interest (arrow or person pointing towards the target, person gazing towards the target object) in the presence of no other cue or a second cue.

As in Experiment 1, longer dwell times are found on gaze and pointing cues compared to arrow cues. The dwell times on arrows are most strongly affected by the presence of pointing cues (and less by the presence of other arrows and gaze cues). When comparing all combinations shown in Figure 8a using a Bonferroni correction, the only significant differences in dwell times on the arrow (towards the target) are found between no cue and the two pointing cues (shorter dwell times on the arrow due to a person pointing in the image) and between no cue and the two arrow cues (less time spent looking at the arrow if there is a second arrow in the image).

The dwell times on gaze cues are more strongly affected by the presence of pointing cues compared to arrows and gaze cues. A significant reduction in the dwell time on the gaze cue is only found between no other cue and a pointing cue away from the target (p< 0.0024).

The dwell times on pointing cues show a mixed picture: arrows also pointing at the target seem to affect the dwell times on the pointing cues less than other types of cues. For the pointing cues, many significant differences are found between pairs of second cues. Adding a second cue leads to a significant drop in dwell times on the pointing cue for all types of other cues (p< 0.0024). Significant drops in dwell times on the pointing cue are also found when replacing the arrow pointing towards the target with any other type of second cue (p< 0.0024).

#### 3.2.2. Dwell Times on the Target

**Figure 9:**
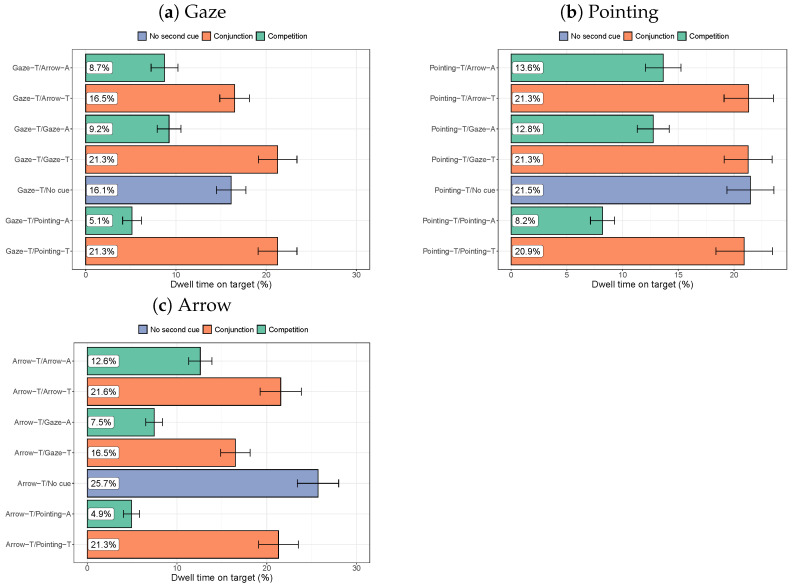
Dwell times on the target (as a percentage of the overall time spent looking at the image). Error bars show the standard error of the mean across participants.

Figure 9 shows the dwell times on targets for the various combinations of cues, split by the cue of interest (an arrow or person pointing at the target, or a person gazing at the target). For gaze cues, the dwell times on the target are longest when there are two cues pointing or looking at the target, with longer dwell times when two people are gazing or pointing at the target (*p* = 0.020 for an extra gaze cue, compared to no cue, and *p* = 0.022 for a second pointing cue). Compared to no second cue, the dwell times on the target are also reduced by an inconsistent cue in the presence of a gaze cue (all *p*-values < 0.001).

For pointing cues, congruent cues do not show an advantage over no second cue. There is a clear cost of an inconsistent cue compared to no second cue (all *p*-values < 0.0013), which is strongest for an inconsistent pointing cue (compared to a person gazing elsewhere or an arrow pointing elsewhere). For arrows, the longest dwell times on the target are found when no second cue is present. This is consistent with the first experiment, where dwell times on the target were reduced by the presence of people in the scene. Dwell times are substantially shorter when one of the cues is pointing or gazing in a different direction from the cue (all *p*-values < 0.001), suggesting that inconsistent cues affect dwell times on targets. Combining a pointing cue towards the target with an arrow towards the target (*p* = 0.10) or two arrows towards the target (*p* = 0.14) did not increase the dwell times on the target compared to not having a second cue.

#### 3.2.3. Time to First Fixation

**Figure 10:**
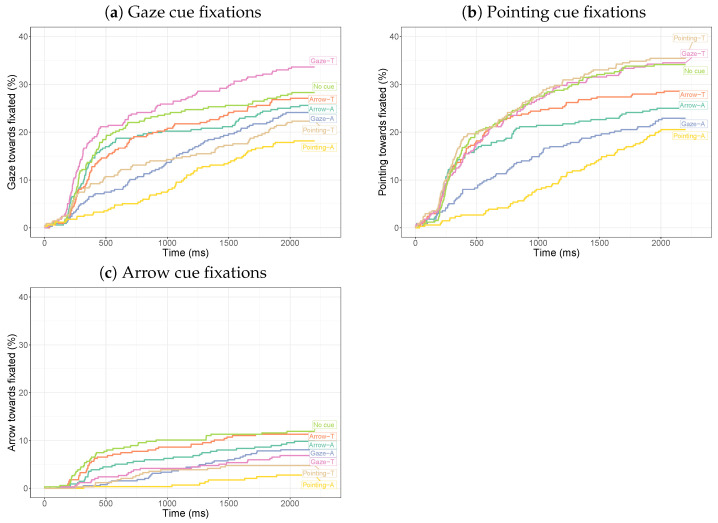
Inverse survival curves for the time until fixation on the cue for the different conditions. For the gaze and pointing cues, the entire cue is used (including body and possibly arm). The focus is on combinations with a gaze cue towards, a pointing cue towards, or an arrow cue towards the target. In constructing the curves, the individual trials were treated as independent measurements.

**Figure 11:**
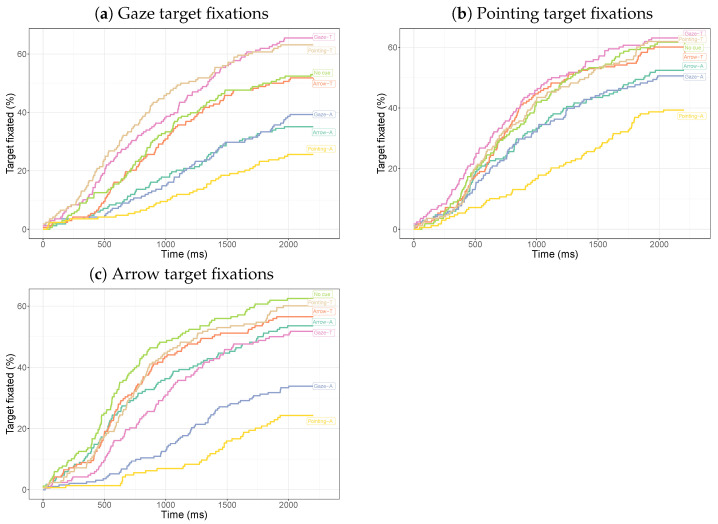
Inverse survival curves for the time until fixation on the target for the different conditions. The focus is on combinations with a gaze cue towards, a pointing cue towards, or an arrow cue towards the target. In constructing the curves, the individual trials were treated as independent measurements.

The time to the first fixation becomes more complicated when there are two cues in the display. We focus on all images with one of the cues pointing towards the target (gaze, pointing, and arrow) and examine the time needed to first fixate this cue towards the target in the presence of another cue or no cue (Figure 10). Likewise, we examine the time needed to first fixate the target with the target cue towards the target and another cue or no cue (Figure 11). For all cues, we observe that a pointing cue draws the observer’s gaze away from the cue and the target more strongly than the other cues (yellow lines in all plots). For all three cues, we see more target fixations than cue fixations. Arrow cues are fixated less than the gaze and pointing cues (Figure 10). Only for arrows are the cue and the target fixated more often when there is no second cue. For gaze and pointing cues, a second gaze or pointing cue towards the target leads to more fixations on the target and the cue itself. Curves hardly intersect, meaning that comparisons for whether a cue or target was fixated yield highly similar results as a comparison of how quickly the cue or target was fixated.

The various curves diverge significantly for fixations on the pointing cue ($\chi^{2}$(6) = 42.6, p< 0.001), the gaze cue ($\chi^{2}$(6) = 31.5, p<0.001), and the arrow cue ($\chi^{2}$(6) = 32.0, p<0.001). Compared to the no-cue conditions, pairwise comparisons with Bonferroni corrections show significantly fewer and later gaze cue fixations for an additional pointing cue away ($\chi^{2}$(1) = 12.4, p<0.001). For pointing cues, significantly fewer and later cue fixations are found with an additional gaze cue away ($\chi^{2}$(1) = 11.8, p<0.001) or a second pointing cue away ($\chi^{2}$(1) = 20.4, p<0.001). For arrows, there are significantly fewer and later cue fixations with an additional pointing cue towards ($\chi^{2}$(1) = 11.9, p<0.001) and an additional pointing cue away ($\chi^{2}$(1) = 20.5, p<0.001) compared to no additional cue.

For fixations on the target, the curves also significantly diverge between conditions for the gaze cue ($\chi^{2}$(6) = 113.9. p<0.001), pointing cue ($\chi^{2}$(6) = 42.9, p<0.001), and arrow cue ($\chi^{2}$(6) = 105.3, p<0.001). Pairwise comparisons with Bonferroni corrections showed that for the gaze cue, the curves for the arrow away ($\chi^{2}$(1) = 12.8, p<0.001), gaze cue away ($\chi^{2}$(1) = 9.95, p<0.001), and pointing cue away ($\chi^{2}$(1) = 31.8, p<0.001) diverged from the no-cue curve. For the pointing cue, only the curve for the additional pointing away cue diverged significantly from the no-cue curve ($\chi^{2}$(1) = 22.9, p<0.001). For the arrow cue, the curves for the gaze away ($\chi^{2}$(1) = 8.69, p<0.001), gaze towards ($\chi^{2}$(1) = 7.45, p<0.001), and pointing towards ($\chi^{2}$(1) = 58.5, p<0.001) cues diverged significantly from the no-cue curve.

#### 3.2.4. Target-Directed Saccades

**Figure 12:**
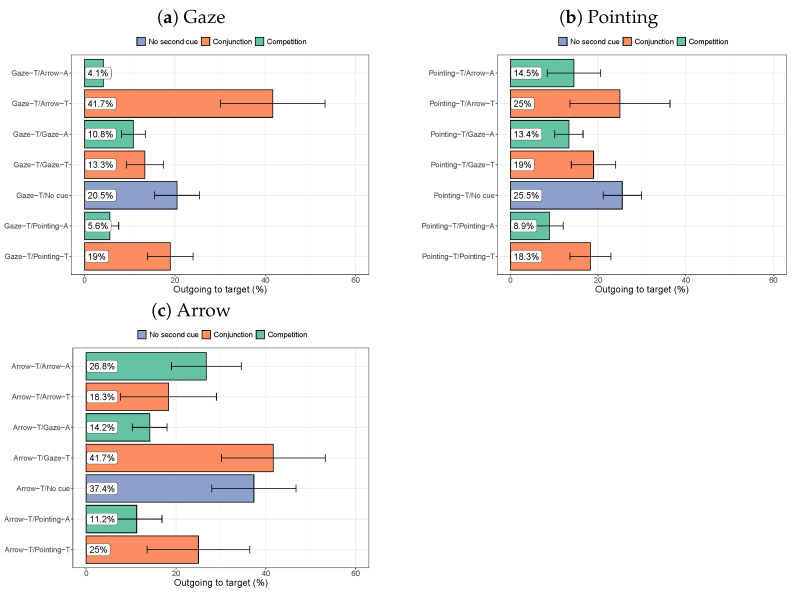
The percentageof saccades that leave the cue of interest and directly land on the target (compared to elsewhere in the display, not returning to the cue of interest). Error bars show the standard error of the mean across participants.

Figure 12 shows the percentage of saccades that leave the cue of interest (an arrow or person pointing at the target or a person gazing at the target) and land on the target (rather than elsewhere in the image). For gaze cueing, the strongest cueing is found when there is also an arrow pointing at the target. The gaze and arrow cues seem to support each other. Only the comparison with the arrow away is the difference from the ‘no cue’ situation significant (*p* = 0.0046).

For the pointing cue, stronger cueing is found when there is another cue pointing at the target than when another cue is pointing elsewhere, but no clear differences are found between the various types of other cues (all *p*-values > 0.014). For arrows, the strongest cueing is found when there is no other cue or when a person is gazing at the target. None of the comparisons, however, reach statistical significance in pairwise comparisons with the ‘no cue’ situation (all *p*-values > 0.05).

### 3.3. Discussion

Experiment 2 investigated the interaction between cues on observers’ eye movements. As in Experiment 1, where single cues were presented, gaze and pointing cues were fixated for longer, more frequently, and earlier than arrow cues. When two cues are presented simultaneously, this preference for looking at people over arrows persists, in line with Experiment 1 and earlier observations [27]. When a scene contained two arrows, the dwell times on the cues were distributed equally across the two arrows. When the second cue was a person, the person attracted longer dwell times than the arrow (in line with earlier findings [27]). People in the scenes who gazed or pointed at the same object as the target of the arrow were gazed at for longer than people gazing or pointing at something else. Simultaneously presented pointing cues away from the target delayed fixations on towards cues and the targets most strongly.

When no other cue was present in the scene, arrows led to longer dwell times on the target than gaze or pointing cues, in line with Experiment 1. A second congruent cue did not always increase the dwell time on the target. A possible reason is that the second cue attracted observers’ overt attention that would have otherwise been directed to the target. A second incongruent cue, however, did reduce overt attention to the target, meaning that these cues not only attracted the observer’s gaze to themselves but also sent the observer’s gaze away from the target. This distraction was strongest for people pointing somewhere other than the target.

Saccades that went from the cue to the target were somewhat affected by other cues in the scene. The variability between participants, however, was large, and many of the comparisons did not reach statistical significance when corrected for the number of comparisons (even when focusing on just the comparison with the ‘no other cue’ situation). It is unfortunate that the results for outgoing saccades were less clear for Experiment 2, as this measure has the advantage that it is less likely to be affected by the position and size of the cue. Within each scene, the target is the same for each of the cues, meaning that the outgoing saccade direction provides a good measure of how strongly the cue guides the next saccade.

Something to take into account when interpreting the data for away cues is that, in our particular setup, these cues did not always point to a target in the scene. Instead, they often pointed to a region inside the image or an object outside the image. This may have affected eye movements away from these cues and may have led to reduced cue following in comparison to towards cues.

## 4. General Discussion

Studies comparing attention shifts induced by social (gaze, pointing) and symbolic (arrows, letters) cues often present cues in isolation and fixation, e.g., [8,41,42]. Here, we extended this work to situations where cues were embedded in natural scenes (by which we mean indoor and outdoor scenes, reflecting the environment where people usually spend time) and not present where participants were already looking by placing the fixation point at one of four positions outside the image so that participants had to saccade into the image [34]. The first experiment replicated the paradigm by Hermens and Walker [31] but with more scenes, more actors, larger arrow cues, and more closely defined regions of interest. The second experiment placed cues in direct competition. In both experiments, participants were asked to ‘simply view’ the images, and fixations were used to measure (overt) attention.

Both experiments showed that observers’ eye movements were more strongly drawn towards people in the scene than towards arrows, in line with previous work [27]. The results also suggest that when an observer’s gaze direction is drawn towards the cue, there is less time available to look at the cued target object, meaning that cues that are fixated less often lead to more time spent looking at the target. In direct competition, people drew the observer’s gaze away from the arrow, but not vice versa. People in the scene also drew the observer’s gaze direction more strongly away from the target when they were pointing or gazing elsewhere in the scene. Arrows led to more direct saccades towards the target when presented in isolation. The competing effects of other cues in the scene on such target-directed saccades directly from the cue were less clear.

Our results with single cues (Experiment 1) showed a difference from the study it was based on [31]. The results were similar in that both studies showed more overt visual attention to people than to arrows in the scene (see also [27]). Differences were found in dwell times on the target and saccades from the cue to the target, with stronger cueing for arrows in the present study. These differences may be caused by the larger arrows in the current study and a difference in the definitions of the regions of interest. In the former study [31], smaller arrow cues were used, as well as bounding boxes around the cues instead of tightly defined regions of interest. The combined effect of these differences means that the size and surface area of the arrow ROI in the scenes was similar between the two studies (as only the arrow region was used in the present study, whereas the entire region around the arrow was used in the past study). For pointing cues, the different methods for defining the regions of interest made a large difference, as a bounding box around a slightly upward or downward arm tends to contain a large portion of the background, which is not the case for a tight region of interest. The present study also segmented the different body parts, meaning that fixations on the head, body, and arm could be compared. This analysis suggested that, possibly unexpectedly, saccades from the body more often went directly to the target than saccades from the pointing arm.

All in all, these differences show that it is important to consider how to define regions of interest. While the past study found no clear differences between tight bounding boxes and slightly wider bounding boxes [31], the move to tight regions of interest does seem to make a difference. Tools developed for labelling within the context of computer vision research, such as LabelMe [43], may aid the labelling of regions of interest for eye movement classifications, as well as tools that can automatically detect heads and bodies [44,45] or automatically segment images into regions [46].

Because we measured eye movements, we can only draw conclusions about overt attention. To examine covert attention, other methods will be needed. For example, a search target could be presented [35], and the influence of cues in the image on search times could be examined. Alternatively, the direction of microsaccades during periods of fixation could be examined, as these have been linked to the direction of covert attention [47,48]. Alternatively, the peak velocity of saccades between fixations could be examined as an indicator of attention [49,50].

In our study, we had young participants (drawn from a student population) and young people as actors (drawn from a pool of researchers and junior staff). A recent study suggested that young participants may be affected more strongly by gaze cues than older participants [32], and it may therefore be interesting to extend the study to an older population, possibly also including older adults as actors. Note that this other study used a search task, while our experiments employed a free-viewing task, and it may therefore be important to compare eye movements between the two types of tasks.

In the present study, we used natural scenes to move away from the use of stimuli presented in isolation and at fixation, which has been shown to elicit stronger cueing effects, particularly for gaze cues, than when observers first have to make a saccade to the cue [25]. We also asked participants to ‘simply view’ the images without any further instructions. At the same time, most of our images contained a cue (arrow, people looking or pointing), which likely revealed our interest in how observers would respond to those cues. The current setup is therefore still quite unlike the ‘real thing’ [51]. Most of the time, people do not ‘simply watch’ a scene. If they watch and see someone point at a target, this is often with another person near the person doing the pointing. More commonly, observers are themselves interacting with the pointing person. If observers look at an arrow sign, it is often because they are looking for directions. Moreover, people do not always simply look, but instead interact with their environment, meaning that they are moving around, touching, and moving objects and interacting with people. In a direct comparison, Foulsham and colleagues [51] compared the viewing patterns of observers when they walked across a university campus and observers who viewed stills from the head-mounted video recordings. Except for a general tendency to fixate the centre of the scene, no clear overlap in viewing patterns was found. Likewise, eye movements towards actual people were different from eye movements towards people shown on a computer screen in the same context [52]. This suggests that further studies are needed that move further towards real-world viewing [26].

Such further studies employing mobile eye tracking will need to work around a few possible issues. First, it is more difficult to present the same stimuli to each participant in the real world. You can work with actors, but you also have to make sure that no one else interferes with your scene. The actors should do the same thing for each participant, but this makes it difficult to actually interact with the participant (as you cannot control what the participant is doing). Second, the analysis of recordings is much more time-consuming, because you will need to define the regions of interest. Deep learning approaches to object detection, such as YOLO [53,54], may aid by providing the bounding boxes around people in the video images needed for a region-of-interest analysis. These bounding boxes suffer from the same issues as the bounding boxes by Hermens and Walker [55], where a large section of the background could be included in the bounding box when actors in the scene point upwards or downwards under an angle. There are algorithms for automatic image segmentation [46], particularly of people in a scene [45], but these approaches are slow and less reliable and may not yet be ready to be used for region detection in mobile eye tracking. Third, creating various scenes in a real-world situation to control for the random effects of being in a particular scene is much more difficult in real-world eye tracking. You may need multiple rooms and to walk participants from one scene to another. Virtual reality may be of assistance here, which may also help in the analysis of the eye-tracking data and the first problem of creating the same scene for each participant, but behaviour or visual attention in virtual reality may differ from in the real world [56,57].

The present results do not directly provide an answer to the question of whether social cues elicit exogenous attention shifts [8,9]. The results indicate that when asked to freely view images, observers first seek out people inside the scene [27,30], prior to fixating arrows. One could argue that these results are in line with an innate people detector, as has been suggested for the eye-gaze direction [12].

Arrows do not seem to attract the observer’s gaze as much as people do, but once observers fixate an arrow, these arrows do steer gaze more strongly in the direction of the cue than the social cues (i.e., saccades that leave the arrow more often go towards the target than saccades that leave social cues). This suggests that arrows direct the observer’s gaze more strongly than rotated heads and bodies and pointing arms, contradicting earlier findings where pointing fingers, arrows, and gaze cues were presented in isolation and at fixation [58] but more in line with those where arrows, rotated heads, pointing fingers, and gaze cues were presented away from fixation [59]. This may suggest that when presented at fixation, arrows and social cues may have different effects than when (initially) presented away from fixation.

To exert their stronger gaze-steering effects, arrows must be fixated first, and it is therefore unclear whether arrows will lead to faster target detection in a visual search task with natural scenes compared to gazing and pointing people, who will be fixated more quickly than arrows. In a practical setting, if you would like to draw someone’s attention to a certain object, people pointing towards this object may therefore still be more effective than an arrow sign.

### Conclusions

The present study compared gaze behaviour towards social and symbolic cues embedded in indoor and outdoor scenes. It replicates earlier observations demonstrating that people draw observers’ attention more strongly than arrows [27,55]. This extra attention to people compared to arrows led to more attention to the cued object in the presence of an arrow. When presented in direct competition, observers preferentially paid attention to people in a scene over arrows, in line with previous work [27]. Simultaneously presented gaze and pointing cues drew attention away from a target cue and its cued object.

## Figures and Tables

**Table 1:** Combinations of cues in Experiment 2 and the number of scenes for each combination.

Combination	Cue 1	Cue 2	Number of Scenes
No cue	-	-	7
One cue	Arrow towards	-	7
	Gaze towards	-	7
	Pointing towards	-	7
Congruent	Arrow towards	Pointing towards	7
	Arrow towards	Gaze towards	7
	Arrow towards	Arrow towards	7
	Gaze towards	Gaze towards	7
	Gaze towards	Pointing towards	7
	Pointing towards	Pointing towards	7
Competition	Arrow towards	Pointing away	7
	Arrow towards	Gaze away	7
	Arrow towards	Arrow away	7
	Arrow away	Pointing towards	7
	Arrow away	Gaze towards	7
	Gaze towards	Gaze away	7
	Pointing towards	Gaze away	7
	Pointing towards	Pointing away	7
	Pointing away	Gaze towards	7

## Data Availability

Fixation data for the two experiments are available from https://osf.io/p763u/ (access on 24 March 2024).
